# Coffee Consumption and Incidence of Cardiovascular and Microvascular Diseases in Never-Smoking Adults with Type 2 Diabetes Mellitus

**DOI:** 10.3390/nu15183910

**Published:** 2023-09-08

**Authors:** Yu-Jie Liu, Meng-Yuan Miao, Jia-Min Wang, Quan Tang, Wen-Wen Han, Yi-Ping Jia, Hao-Wei Tao, Yan Zheng, Rob M. van Dam, Li-Qiang Qin, Guo-Chong Chen

**Affiliations:** 1 Department of Nutrition and Food Hygiene, Suzhou Medical College of Soochow University, Suzhou 215127, China; 20215247014@stu.suda.edu.cn (Y.-J.L.); mmybbyxy@163.com (M.-Y.M.); 20225247067@stu.suda.edu.cn (J.-M.W.); hanww0307@163.com (W.-W.H.); 20225247039@stu.suda.edu.cn (Y.-P.J.); thw13770101999@163.com (H.-W.T.); 2 Yancheng Center for Disease Control and Prevention, Yancheng 224002, China; tangquan_2008@126.com; 3 State Key Laboratory of Genetic Engineering, Human Phenome Institute, School of Life Sciences, Fudan University, Shanghai 200438, China; yan_zheng@fudan.edu.cn; 4 Ministry of Education Key Laboratory of Public Health Safety, School of Public Health, Fudan University, Shanghai 200032, China; 5 National Clinical Research Center for Aging and Medicine, Huashan Hospital, Fudan University, Shanghai 200040, China; 6 Department of Exercise and Nutrition Sciences, Milken Institute School of Public Health, The George Washington University, Washington, DC 20037, USA; rob.van.dam@nus.edu.sg

**Keywords:** coffee, cardiovascular diseases, microvascular diseases, type 2 diabetes mellitus

## Abstract

The relationship between coffee consumption and diabetes-related vascular complications remains unclear. To eliminate confounding by smoking, this study assessed the relationships of coffee consumption with major cardiovascular disease (CVD) and microvascular disease (MVD) in never-smokers with type 2 diabetes mellitus (T2DM). Included were 9964 never-smokers with T2DM from the UK Biobank without known CVD or cancer at baseline (7781 were free of MVD). Participants were categorized into four groups according to daily coffee consumption (0, 0.5–1, 2–4, ≥5 cups/day). CVD included coronary heart disease (CHD), myocardial infarction (MI), stroke, and heart failure (HF). MVD included retinopathy, peripheral neuropathy, and chronic kidney disease (CKD). Cox regression models were used to estimate hazard ratios (HRs) and 95% confidential intervals (CIs) of total CVD and MVD and the component outcomes associated with coffee consumption. During a median of 12.7 years of follow-up, 1860 cases of CVD and 1403 cases of MVD were identified. Coffee intake was nonlinearly and inversely associated with CVD (P-nonlinearity = 0.023) and the component outcomes. Compared with no coffee intake, HRs (95% CIs) associated with a coffee intake of 2 to 4 cups/day were 0.82 (0.73, 0.93) for CVD, 0.84 (0.73, 0.97) for CHD, 0.73 (0.57, 0.92) for MI, 0.76 (0.57, 1.02) for stroke, and 0.68 (0.55, 0.85) for HF. Higher coffee intake (≥5 cups/day) was not significantly associated with CVD outcomes. Coffee intake was linearly and inversely associated with risk of CKD (HR for ≥5 vs. 0 cups/day = 0.64; 95% CI: 0.45, 0.91; P-trend = 0.0029) but was not associated with retinopathy or peripheral neuropathy. Among never-smoking individuals with T2DM, moderate coffee consumption (2–4 cups/day) was associated with a lower risk of various CVD outcomes and CKD, with no adverse associations for higher consumption.

## 1. Introduction

Type 2 diabetes mellitus (T2DM) is a significant public health concern affecting approximately 6059 per 100,000 persons worldwide in 2017, and this estimate is projected to reach 7079 per 100,000 by 2030 [1]. The disease progression of T2DM is commonly accompanied by the emergence of macrovascular complications (i.e., coronary heart disease (CHD), myocardial infarction (MI), stroke, and heart failure (HF)) and microvascular complications (i.e., retinopathy, peripheral neuropathy, and chronic kidney disease (CKD)) [2,3,4,5]. Therefore, identifying modifiable risk factors for macrovascular and microvascular complications is crucial for the management of T2DM.

Coffee is a widespread beverage throughout the world, such that even small effects of coffee consumption on health risks could have significant public health consequences. Over the past few decades, a large body of epidemiologic evidence has linked habitual coffee consumption to a lower risk of various health outcomes [6], including T2DM [7]. With regard to vascular conditions, moderate coffee consumption has been linked to a reduced likelihood of developing cardiovascular disease (CVD) [8] and specific microvascular diseases (MVD) such as CKD [9] in the general population. Among individuals with T2DM, the relationship between coffee consumption and diabetes-related vascular complications has been less understood [10,11,12].

It is noteworthy that coffee consumption usually has a strong correlation with tobacco smoking [13,14]. Given the strong impact of smoking on various health outcomes, the inadequate adjustment for smoking could attenuate inverse associations (or exaggerate direct associations) between coffee consumption and health outcomes. We therefore leveraged data from the UK Biobank study to investigate the relationships of coffee consumption with the long-term risk of developing CVD and MVD among individuals with T2DM. The large sample size of the UK Biobank allows for limiting the analysis to never-smoking adults with T2DM, thereby eliminating confounding by smoking and obtaining a more accurate measure of the relationships between coffee consumption and risk of CVD and MVD.

## 2. Materials and Methods

### 2.1. Study Population

The UK Biobank study has been previously documented with comprehensive elucidations of its design, methodologies, and participants involved [15]. Between 2006 and 2010, this study successfully enlisted approximately 500,000 participants aged between 37 and 73 years from 22 assessment centers dispersed across England, Scotland, and Wales. At baseline, information regarding demographic and socioeconomic factors, lifestyle, physical measures, and health-related data was collected. Approval for this study was granted by the North West Multicenter Research Ethics Committee (approval letter dated 17 June 2011, Ref 11/NW/0382), and all participants provided written consent after being fully informed.

For the present study, never-smoking adults who had T2DM at baseline were considered as potentially eligible participants. As reported elsewhere [16], T2DM was considered present according to one or more of the following 4 diagnostic criteria: (1) self-reported medical history and medication use; (2) blood levels of glycated hemoglobin (HbA1c) ≥6.5% (48 mmol/mol); (3) hospital inpatient records; and (4) not type 1 diabetes mellitus at baseline. Participants who met the following criteria were included: (1) had a history of T2DM; (2) self-reported as never-smokers; (3) had information on coffee consumption; and (4) had no history of CVD or cancer. Participants who had prevalent MVD were further excluded when assessing the relationship of coffee intake with incident MVD outcomes. Finally, we included 9964 never-smoking adults with T2DM, among whom 7781 were free of MVD (Supplementary Figure S1).

### 2.2. Assessment of Coffee Consumption

Self-reported data on coffee consumption at baseline were collected using a touchscreen-based questionnaire. Participants were asked to report the number of cups of coffee (integer or 0.5 if less than one) they consumed per day using the following question: “Please indicate the average number of cups of coffee you consume per day, including decaffeinated coffee”. Participants who reported consuming more than 10 cups per day were requested to reconfirm their response. Additionally, coffee drinkers were further asked to select the type of coffee they usually drink (decaffeinated, instant, ground, or other types of coffee).

### 2.3. Incident Cardiovascular and Microvascular Outcomes

Primary outcomes of interest were incident total CVD, which was a composite indicator of the first occurrence of CHD, MI, stroke, or HF; and incident total MVD, which was defined as new-onset retinopathy, peripheral neuropathy, or CKD during follow-up. The secondary outcomes were individual CVD and MVD as listed above. Consistent with prior studies on CVD or MVD [17,18,19], we determined the outcomes based on cumulative medical records of hospital diagnoses and identified the incident cases according to the International Classification of Disease (ICD), 10th version (ICD-10). The definitions of CVD and MVD in the UK biobank are provided in Supplementary Table S1.

### 2.4. Assessment of Other Covariates

Participants provided information on sociodemographic characteristics, lifestyle factors, and medical histories through touchscreen questionnaires. Townsend deprivation index was generated using information on unemployment, non-car ownership, non-home ownership, and household overcrowding. Physical activity was assessed using the self-reported short-form international physical activity questionnaire and the data are summarized and reported in MET-h per week. A healthy diet score was calculated based on the following 6 dietary habits [20]—vegetables and fruit: fresh vegetables ≥3 servings/day or fresh fruit ≥3 servings/day or a combination ≥4.5 servings/day; fish ≥2 servings/week; whole grains ≥3 servings/week; refined grains <1.5 servings/week; red meat <2 servings/week; and processed meat <1 serving/week. One point was awarded for each favorable dietary habit and a higher dietary score represents healthier dietary habits. BMI was calculated using measured weight and height (kg/m^2^). Baseline hypertension was defined as systolic blood pressure ≥140 mmHg, diastolic blood pressure ≥90 mmHg, or self-reported physician’s diagnosis or medication use. Hyperlipidemia was defined as self-reported physician’s diagnosis or use of lipid-lowering medications. Duration of T2DM was calculated by subtracting a participant’s age at diagnosis of T2DM from the age at baseline interview; and for T2DM cases that were identified using HbA1c only, the duration was deemed to be zero years [21]. HbA1c levels were quantified through high-performance liquid chromatography (Variant II, Bio-Rad Laboratories).

### 2.5. Statistical Analysis

Baseline participant characteristics are reported according to categories of daily coffee consumption (0, 0.5–1, 2–4, and ≥5 cups/day), as numbers (percentages) or mean ± SDs where appropriate. Cox regression models were used to estimate hazard ratios (HRs) and 95% confidential intervals (CIs) of CVD, MVD, and the component outcomes associated with different levels of coffee consumption, using 0 cups per day as the reference group. Person-time of follow-up was calculated as the duration from the date of baseline evaluation until the date of the diagnosis of CVD/MVD, death, loss to follow-up, or end of follow-up, whichever occurred first. Three models with an increasing degree of adjustment for potential confounders were developed. Model 1 was adjusted for age, sex, race and ethnicity (White, Asian or Asian British, Black or Black British, and mixed), and Townsend deprivation index (in quintile). Model 2 was further adjusted for BMI (kg/m^2^), physical activity (MET-h/week), alcohol consumption (never, former, current: <1, 1–2, and ≥3 drinks/week), and the dietary score (points). Model 3 included all covariates in model 2 with an additional adjustment for hypertension (yes, no), hyperlipidemia (yes, no), HbA1c levels (mmol/mol), and diabetes duration (years).

To assess the potential nonlinearity for the assessed relationships, we plotted restricted cubic splines with 5 knots (at 5th, 27.5th, 50th, 72.5th, and 95th percentiles) by treating daily coffee consumption as a continuous variable. Stratified analyses were performed to assess potential difference in the examined associations according to the following participant characteristics: age (<60 or ≥60 y), sex, ethnicity (white or non-white), Townsend deprivation index (<0 or ≥0), current alcohol drinking (0, ≤2 drinks/week, or >2 drinks/week), BMI (<30 or ≥30 kg/m^2^), HbA1c (<7.0% or ≥7.0%), physical activity (meet the recommended levels or not), diet score (<3 or ≥3), hypertension (yes or no), and hyperlipidemia (yes or no). *p* values for interactions were calculated using likelihood ratio test.

Finally, we performed an additional analysis that considered the usual type of coffee the participants consumed. Specifically, we compared the risk of CVD/MVD for participants with different levels of coffee consumption (0.5–1, 2–4, and ≥5 cups/day) with coffee non-consumers, for participants who usually drank decaffeinated, instant, or ground coffee. Statistical analyses were performed using R (version 4.2.3) or SAS (version 9.4). A two-sided *p* value of <0.05 was considered statistically significant.

## 3. Results

### 3.1. Participant Characteristics

Of the 9964 never-smoking participants with T2DM (mean (SD) age was 57.9 (7.6) years), 2859 were coffee non-consumers, and 2859, 2779, 3449, and 877 participants reported a coffee consumption of 0.5 to 1, 2 to 4, and 5 or more cups per day, respectively.

Overall, when compared with individuals with no coffee consumption, those who consumed coffee (regardless of amounts) were more likely to be male, ethnically white, and current alcohol consumers; had a lower Townsend deprivation index (higher socioeconomic status); were more likely to have hyperlipidemia; and were less likely to have prevalent retinopathy or CKD. In addition, participants who consumed 5 or more cups per day of coffee (but not lower levels of coffee consumption), as compared with those with no coffee consumption, had higher BMI and HbA1c levels, lower physical activity, a lower dietary score, and a longer duration of diagnosis of T2DM (Table 1).

### 3.2. Associations of Coffee Consumption with Cardiovascular and Microvascular Diseases

During a median follow-up of 12.7 years, 1860 incident cases of CVD (including 1411 CHD, 552 MI, 329 stroke, and 539 HF cases) and 1403 incident cases of MVD (including 803 retinopathy, 191 peripheral neuropathy, and 631 CKD cases) were identified.

**Figure 1:**
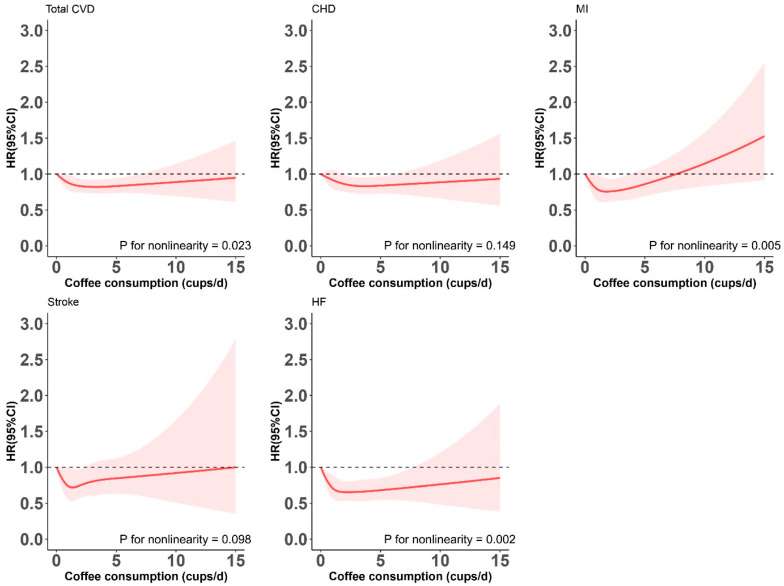
Restricted cubic splines for the relationships between coffee consumption and risk of total and individual cardiovascular diseases among never-smoking adults with T2DM. CHD, coronary heart disease; CVD, cardiovascular disease; HF, heart failure; MI, myocardial infarction. The splines were modeled with five knots (5th, 27.5th, 50th, 72.5th, and 95th percentiles), and the level of 0 cups/day was used as the reference. Results were adjusted for age (continuous, years), sex, ethnicity (White, Asian or Asian British, Black or Black British, and mixed), Townsend deprivation index (in quintile), BMI (continuous, kg/m^2^), physical activity (continuous, MET-h/week), alcohol consumption (never, former, current: <1, 1–2, and ≥3 drinks/week), diet score (continuous, points), hypertension (yes, no), hyperlipidemia (yes, no), HbA1c levels (continuous, mmol/mol), and diabetes duration (continuous, years). Note: The red areas around the spline curves represent the 95% confidence intervals.

After multivariable adjustment, there was a nonlinear inverse association between coffee consumption and risk of total CVD (P-nonlinearity = 0.023) (Figure 1). As compared with no coffee consumption, the fully adjusted HRs (95% CI) of total CVD were 0.91 (0.80, 1.04), 0.82 (0.73, 0.93), and 0.89 (0.74, 1.07) for 0.5 to 1, 2 to 4, and 5 or more cups per day of coffee consumption, respectively (Table 2). The patterns of the associations for individual CVD outcomes were similar to that for total CVD, with the lowest risk observed at 2 to 4 cups of coffee per day (Figure 1). When compared with no coffee consumption, HRs (95% CI) associated with 2 to 4 cups of coffee per day were 0.84 (0.73, 0.97) for CHD, 0.73 (0.57, 0.92) for MI, 0.76 (0.57, 1.02) for stroke, and 0.68 (0.55, 0.85) for HF. A coffee consumption of 5 or more cups per day was not significantly associated with CVD outcomes (Table 2).

**Figure 2:**
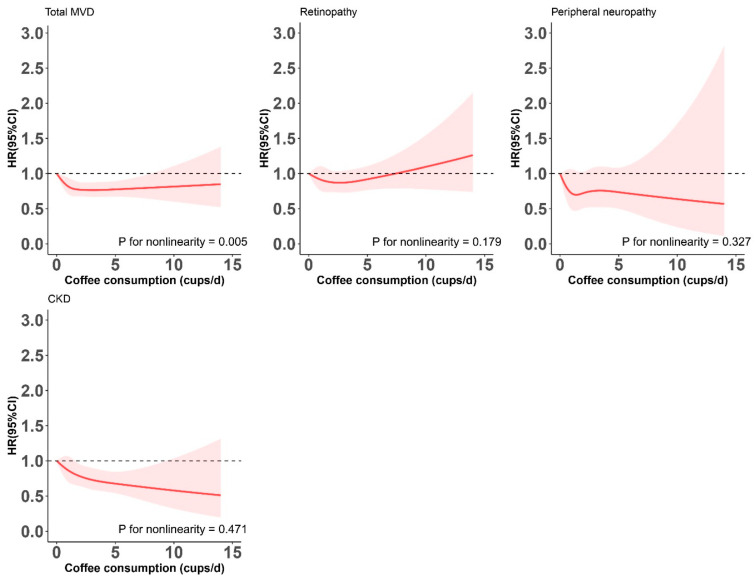
Restricted cubic splines for the relationships between coffee consumption and risk of total and individual microvascular diseases among never-smoking adults with T2DM. CKD, chronic kidney disease; MVD, microvascular disease. The splines were modeled with five knots (5th, 27.5th, 50th, 72.5th, and 95th percentiles), and the level of 0 cups/day was used as the reference. Results were adjusted for age (continuous, years), sex, ethnicity (White, Asian or Asian British, Black or Black British, and mixed), Townsend deprivation index (in quintile), BMI (continuous, kg/m^2^), physical activity (continuous, MET-h/week), alcohol consumption (never, former, current: <1, 1–2, and ≥3 drinks/week), diet score (continuous, points), hypertension (yes, no), hyperlipidemia (yes, no), HbA1c levels (continuous, mmol/mol), and diabetes duration (continuous, years). Note: The red areas around the spline curves represent the 95% confidence intervals.

Coffee intake was inversely associated with risk of total MVD in a nonlinear manner (P-nonlinearity = 0.005) (Figure 2), with fully adjusted HRs (95% CI) of 0.89 (0.77, 1.03), 0.81 (0.70, 0.94), and 0.81 (0.65, 0.99) for 0.5 to 1, 2 to 4, and 5 or more cups of coffee per day, respectively, compared with no coffee consumption. This inverse association appeared to be largely driven by the association of coffee consumption with CKD (HR ≥ 5 vs. 0 cups/day = 0.64; 95% CI: 0.45–0.91; P-trend = 0.003), whereas no associations were found for retinopathy (P-trend = 0.782) or peripheral neuropathy (P-trend = 0.431) (Table 3).

### 3.3. Subgroup Analysis

Given the nonlinear association of coffee consumption with CVD, the stratified analyses were performed to examine moderate coffee consumption (2 to 4 cups per day, vs. no coffee intake) in relation to risk of CVD. For CKD, results were reported for each additional increment of two cups per day. The inverse associations of moderate coffee intake with risk of CVD, or increment coffee intake with risk of CKD were broadly consistent across the population subgroups predefined by various sociodemographic characteristics, lifestyle factors, and medical histories (all P-interaction > 0.05) (Supplementary Figure S2).

### 3.4. Coffee Consumption, Coffee Type, and Cardio- and Microvascular Diseases

As shown in Supplementary Figure S3 and Supplementary Table S2, consumption of 2 to 4 cups of coffee per day (vs. no coffee consumption) was significantly associated with a lower risk of total CVD for participants who usually consumed instant coffee (HR = 0.80; 95% CI: 0.69, 0.92) or ground coffee (HR = 0.77; 95% CI: 0.62, 0.96) but not for decaffeinated-coffee consumers (HR = 0.93; 95% CI: 0.76, 1.12). A coffee intake of 2 to 4 cups/day was associated with a lower risk of total MVD among those who usually consumed decaffeinated coffee (HR = 0.71; 95% CI: 0.56, 0.90) or instant coffee (HR = 0.85; 95% CI: 0.72, 0.99) with a similar but non-significant association among ground-coffee consumers (HR = 0.84; 95% CI: 0.66, 1.07). These associations varied when considering individual CVD and MVD outcomes (Supplementary Table S2).

Participants with a coffee consumption of 5 or more cups per day (vs. no coffee consumption) had a lower risk of CKD (HR = 0.34; 95% CI: 0.14, 0.82) only among those who usually drank decaffeinated coffee, without a significantly altered risk for any other CVD or MVD outcomes irrespective of the types of coffee consumed (Supplementary Table S2).

## 4. Discussion

In this prospective study of never-smoking adults with T2DM, we investigated coffee consumption in relation to total CVD and MVD and their component outcomes. Our findings showed that a moderate coffee consumption of 2 to 4 cups per day (vs. no coffee consumption) was associated with a lower risk of total CVD and individual CVD outcomes. Higher levels of coffee consumption (≥5 cups/day) were not significantly associated with a risk of total or any individual CVD outcomes. A higher coffee consumption was linearly associated with a lower risk of CKD, but was not associated with retinopathy or peripheral neuropathy.

Residual confounding by smoking is often a concern for the studies of coffee consumption and health outcomes. In general, coffee consumption is strongly associated with cigarette smoking and evidence from Mendelian randomization analysis supports a causal link between smoking and coffee drinking [13]. A study of two large cohorts of US health professionals with T2DM [22] found that merely 10.2% of individuals who consumed less than one cups/month were current smokers, whereas the corresponding figure for those consuming ≥4 cups/day of coffee was 35.3%. For current smokers, coffee consumption tends to be associated with a larger amount [23] and longer duration of smoking [24]; all these coffee-related smoking features are linked to an elevated risk of diabetes-related vascular complications [25]. In this context, an inadequate adjustment for smoking may attenuate an inverse association (or exaggerate a positive association) between coffee consumption and health risks. Therefore, to completely eliminate confounding by smoking and gain a more accurate estimation of the relationships between coffee intake and risk of CVD/MVD, our current analysis was conducted among individuals with T2DM who were never-smokers.

An Inverse relationship of coffee consumption with risk of CVD has been widely reported in previous prospective studies conducted in the general population [8]. Only a few studies have been focused on the coffee–CVD association among individuals with T2DM [12,22]. In a recent meta-analysis combining data from two cohort studies (1093 CVD events among 10,667 individuals with T2DM), each 4-cup-per-day increment in coffee consumption was significantly associated with a 33% lower risk of total CVD [12], which is in line with recent findings from a pooled analysis of US health professionals [22].

Results from a Mendelian randomization analysis support a beneficial influence of coffee intake on kidney function [26]. In the general population, an inverse association between coffee intake and risk of CKD has been reported [9], whereas data for retinopathy or peripheral neuropathy are very limited. A systematic review and meta-analysis of four cohort studies with 25,849 participants found that coffee consumers had a significant 13% lower risk of incident CKD as compared with non-consumers [9]. Among individuals with T2DM, only a few studies have examined the relationship of coffee consumption with the risk of decline in kidney function [11] or retinopathy [10]. An analysis of data from the Fukuoka Diabetes Registry showed that coffee consumption was associated with a significantly lower risk of decline in kidney function in patients with T2DM [11]. In a cross-sectional analysis of 1350 participants from the 2008–2011 Korean National Health and Nutrition Survey, coffee consumption was inversely associated with the prevalence of diabetic retinopathy [10]. We found that, among never-smokers with T2DM, higher coffee consumption was linearly associated with a lower risk of CKD, but was not associated with retinopathy or peripheral neuropathy.

Coffee contains many bioactive components such as caffeine, phenolic compounds, lignans, and trigonelline [27]. Phenolic compounds in coffee (e.g., phenol chlorogenic acid) have been demonstrated to stimulate secretion of glucagon-like peptide-1, which may thereby improve glucose-induced insulin secretion and insulin action [28,29]. It is estimated that, on average, each cup (~200 mL) of coffee has about 100 mg of caffeine and 350 mg of phenolic compounds [30]. Moreover, coffee consumption is associated with a lower level of proinflammatory agents [31,32] and higher levels of anti-inflammatory biomarkers [33]. Recent studies have also identified and validated several coffee-related blood metabolites to be associated with incident CKD [34]. These putative biological actions of coffee components may jointly underlie the observed inverse associations between moderate coffee consumption and risk of CVD and CKD.

On the other hand, short-term metabolic studies have shown that acute caffeine intake results in elevated blood pressure through sympathetic activation, but tolerance develops quickly and there are minimal effects on long-term blood pressure control [35,36]. In addition, cafestol in unfiltered coffee increases serum total cholesterol concentrations [30]. However, the lack of association between relatively high coffee consumption (≥5 cup/day) and risk of CVD in the current study is unlikely explained by these potential influences of coffee components on blood pressure or serum lipids, but may be attributable, at least in part, to residual confounding. In the current study, participants who consumed 5 or more cups of coffee per day as compared with those with no coffee consumption had a longer diabetes duration and a poorer risk profile including higher BMI and HbA1c and lower physical activity and diet quality. Furthermore, it is also possible that individuals who consumed high levels of coffee are those who prefer adding sugar, artificial sweeteners, and milk to coffee based on their personal preferences [37]. Actually, the UK biobank completed five rounds of 24 h questionnaires that specified not only the type of coffee but whether milk and/or sweeteners were added. However, only a small proportion of participants completed the 24 h questionnaire at BASELINE (2009–2010), which is insufficient to provide more robust evidence for our analysis of never-smoking adults with T2DM, and so we did not consider these data for our analysis.

As far as we know, the present study is the first to examine the associations of coffee consumption with incident CVD and MVD and their component outcomes among individuals with T2DM who have never smoked. Thus, a key strength of the present study is the elimination of confounding by smoking. Moreover, our analysis was conducted in a population-based cohort, which included a large sample size of T2DM participants with a wide age range who were followed-up for a long period. Additional strengths of the present study include the comprehensive adjustment for other lifestyle and clinical features including glycemic control and duration of diagnosis of T2DM. Several limitations to our study should be acknowledged. First, as mentioned above, the influence of residual or unmeasured confounding (including confounding by sugar, artificial sweeteners, or milk added to coffee) on our findings cannot be fully excluded, especially for the findings observed for high levels of coffee consumption. Second, data on daily coffee intake were assessed using a self-reported questionnaire at baseline. As a result, neither potential response bias nor changes in coffee consumption during the follow-up period were taken into account. Third, the questionnaire did not assess the intake of different types of coffee, but instead asked about the type of coffee the participants usually consumed. Thus, dose–response relationships between the consumption of specific types of coffee and risk of CVD/MVD are yet to be assessed in future studies. Finally, a high proportion of participants in the present study were white UK residents, which may limit the generalizability of our findings to other racial/ethnic populations.

## 5. Conclusions

To summarize, in a prospective study of never-smoking adults with T2DM, our findings suggest that moderate coffee consumption (2–4 cups/day) was associated with a lower risk of total and individual cardiovascular diseases and chronic kidney disease, with no adverse associations for higher coffee consumption.

## Figures and Tables

**Table 1:** Baseline participant characteristics according to coffee consumption.

	Total	Coffee Consumption (cups/day)			
	N = 9964	None (n = 2859)	0.5–1 (n = 2779)	2–4 (n = 3449)	≥5 (n = 877)
Age, y	57.9 ± 7.6	56.9 ± 7.8	58.3 ± 7.5	58.4 ± 7.4	57.9 ± 7.5
Men	4948 (49.7)	1269 (44.4)	1381 (49.7)	1785 (51.8)	513 (58.5)
White	7889 (79.2)	1889 (66.1)	2122 (76.4)	3041 (88.2)	837 (95.4)
Townsend deprivation index ^a^	−0.55 ± 3.4	0.09 ± 3.5	−0.52 ± 3.4	−0.98 ± 3.3	−1.07 ± 3.1
BMI, kg/m^2^					
<25	1239 (12.5)	358 (12.5)	404 (14.5)	398 (11.5)	79 (9.0)
25 to <30	3437 (34.5)	1008 (35.3)	972 (35.0)	1185 (34.4)	272 (31.0)
≥30	5288 (53.0)	1493 (52.2)	1403 (50.5)	1866 (54.1)	526 (60.0)
Physical activity, MET-h/week	38.4 ± 37.7	38.8 ± 37.7	38.9 ± 38.6	38.1 ± 37.0	36.9 ± 37.4
Alcohol consumption					
Never	1476 (14.8)	743 (26.0)	353 (12.7)	311 (9.0)	69 (7.9)
Former	539 (5.4)	214 (7.5)	120 (4.3)	162 (4.7)	43 (4.9)
Current: <1 drinks/week	3333 (33.5)	951 (33.3)	991 (35.7)	1097 (31.8)	294 (33.5)
Current: 1–2 drinks/week	2265 (22.7)	521 (18.2)	654 (23.5)	877 (25.4)	213 (24.3)
Current: ≥3 drinks/week	2339 (23.5)	423 (14.8)	659 (23.7)	1000 (29.0)	257 (29.3)
Diet score ≥3 points	5550 (55.7)	1612 (56.4)	1596 (57.4)	1900 (56.1)	442 (50.4)
Hypertension	7824 (78.5)	2192 (76.7)	2205 (79.4)	2747 (79.7)	680 (77.5)
Hyperlipidemia	6224 (62.5)	1670 (58.4)	1725 (62.1)	2227 (64.6)	602 (68.6)
HbA1c, mmol/L	52.7 ± 14.6	52.7 ± 15.0	52.7 ± 14.2	52.4 ± 14.4	53.8 ± 15.5
Diabetes duration, y					
0–<5	5746 (57.7)	1701 (59.5)	1617 (58.2)	1984 (57.5)	444 (50.6)
5–<10	2181 (21.9)	572 (20.0)	604 (21.7)	788 (22.9)	217 (24.7)
≥10	2037 (20.4)	586 (20.5)	558 (20.1)	677 (19.6)	216 (24.6)
Retinopathy	1652 (16.6)	492 (17.2)	474 (17.1)	553 (16.0)	133 (15.2)
Peripheral neuropathy	92 (0.90)	28 (0.98)	22 (0.79)	30 (0.87)	12 (1.37)
CKD	643 (6.5)	209 (7.3)	171 (6.2)	215 (6.2)	48 (5.5)

BMI, body mass index; CKD, chronic kidney disease; NA, not applicable. ^a^ A higher Townsend deprivation index indicates a greater degree of deprivation (or lower socioeconomic status).

**Table 2:** Hazard ratios of total and individual cardiovascular diseases associated with coffee consumption.

	Coffee Consumption (cups/day)				P for Trend	P for Nonlinearity
	None	0.5–1	2–4	≥5		
Total CVD						
Events/N	548/2859	528/2779	600/3449	184/877		
Model 1	1.00 (Ref.)	0.92 (0.82, 1.04)	0.83 (0.74, 0.93)	1.01 (0.85, 1.19)	0.252	
Model 2	1.00 (Ref.)	0.95 (0.84, 1.07)	0.83 (0.74, 0.94)	0.96 (0.80, 1.14)	0.061	
Model 3	1.00 (Ref.)	0.91 (0.80, 1.04)	0.82 (0.73, 0.93)	0.89 (0.74, 1.07)	0.037	0.023
CHD						
Events/N	412/2859	410/2779	454/3449	135/877		
Model 1	1.00 (Ref.)	0.96 (0.84, 1.10)	0.84 (0.74, 0.96)	0.98 (0.81, 1.19)	0.232	
Model 2	1.00 (Ref.)	0.98 (0.85, 1.13)	0.85 (0.74, 0.97)	0.94 (0.76, 1.15)	0.162	
Model 3	1.00 (Ref.)	0.93 (0.80, 1.07)	0.84 (0.73, 0.97)	0.85 (0.69, 1.06)	0.052	0.149
MI						
Events/N	174/2859	151/2779	162/3449	65/877		
Model 1	1.00 (Ref.)	0.83 (0.67, 1.03)	0.71 (0.57, 0.88)	1.10 (0.83, 1.47)	0.752	
Model 2	1.00 (Ref.)	0.85 (0.68, 1.08)	0.73 (0.58, 0.91)	1.06 (0.78, 1.43)	0.551	
Model 3	1.00 (Ref.)	0.82 (0.64, 1.04)	0.73 (0.57, 0.92)	1.06 (0.78, 1.45)	0.735	0.005
Stroke						
Events/N	100/2859	90/2779	102/3449	37/877		
Model 1	1.00 (Ref.)	0.86 (0.64, 1.14)	0.78 (0.59, 1.03)	1.14 (0.78, 1.66)	0.857	
Model 2	1.00 (Ref.)	0.94 (0.70, 1.27)	0.82 (0.61, 1.10)	1.16 (0.78, 1.72)	0.819	
Model 3	1.00 (Ref.)	0.82 (0.61, 1.10)	0.76 (0.57, 1.02)	0.98 (0.65, 1.48)	0.378	0.098
HF						
Events/N	177/2859	144/2779	166/3449	52/877		
Model 1	1.00 (Ref.)	0.77 (0.62, 0.96)	0.71 (0.57, 0.88)	0.89 (0.65, 1.21)	0.182	
Model 2	1.00 (Ref.)	0.76 (0.61, 0.96)	0.66 (0.53, 0.82)	0.75 (0.54, 1.03)	0.054	
Model 3	1.00 (Ref.)	0.80 (0.64, 1.01)	0.68 (0.55, 0.85)	0.75 (0.54, 1.05)	0.022	0.002

CHD, coronary heart disease; CVD, cardiovascular disease; HF, heart failure; MI, myocardial infarction. Model 1 was adjusted for age (continuous, years), sex, ethnicity (White, Asian or Asian British, Black or Black British, and mixed), and Townsend deprivation index (in quintile). Model 2 was further adjusted for BMI (continuous, kg/m^2^), physical activity (continuous, MET-h/week), alcohol consumption (never, former, current: <1, 1–2, and ≥3 drinks/week), and diet score (continuous, points). Model 3 included all covariates from Model 2, plus an additional adjustment for hypertension (yes, no), hyperlipidemia (yes, no), HbA1c levels (continuous, mmol/mol), and diabetes duration (continuous, years).

**Table 3:** Hazard ratios of total and individual microvascular diseases associated with coffee consumption.

	Coffee Consumption (cups/day)				P for Trend	P for Nonlinearity
	None	0.5–1	2–4	≥5		
Total MVD						
Events/N	426/2191	393/2177	463/2716	121/697		
Model 1	1.00 (Ref.)	0.86 (0.75, 0.98)	0.81 (0.71, 0.92)	0.84 (0.68, 1.03)	0.075	
Model 2	1.00 (Ref.)	0.87 (0.76, 0.99)	0.80 (0.70, 0.92)	0.82 (0.67, 1.02)	0.053	
Model 3	1.00 (Ref.)	0.89 (0.77, 1.03)	0.81 (0.70, 0.94)	0.81 (0.65, 0.99)	0.045	0.005
Retinopathy						
Events/N	230/2191	225/2177	269/2716	79/697		
Model 1	1.00 (Ref.)	0.94 (0.78, 1.13)	0.90 (0.75, 1.08)	1.07 (0.82, 1.38)	0.733	
Model 2	1.00 (Ref.)	0.96 (0.80, 1.17)	0.90 (0.75, 1.08)	1.07 (0.82, 1.39)	0.751	
Model 3	1.00 (Ref.)	0.98 (0.80, 1.20)	0.90 (0.73, 1.09)	1.05 (0.80, 1.38)	0.782	0.179
Peripheral neuropathy						
Events/N	64/2191	46/2177	67/2716	14/697		
Model 1	1.00 (Ref.)	0.72 (0.49, 1.05)	0.84 (0.60, 1.19)	0.68 (0.38, 1.21)	0.432	
Model 2	1.00 (Ref.)	0.69 (0.47, 1.03)	0.82 (0.57, 1.17)	0.63 (0.34, 1.15)	0.392	
Model 3	1.00 (Ref.)	0.72 (0.47, 1.09)	0.82 (0.56, 1.20)	0.65 (0.35, 1.19)	0.431	0.327
CKD						
Events/N	192/2191	188/2177	205/2716	46/697		
Model 1	1.00 (Ref.)	0.90 (0.73, 1.10)	0.77 (0.63, 0.94)	0.69 (0.50, 0.96)	0.009	
Model 2	1.00 (Ref.)	0.94 (0.76, 1.15)	0.79 (0.64, 0.98)	0.68 (0.49, 0.95)	0.006	
Model 3	1.00 (Ref.)	0.95 (0.76, 1.18)	0.77 (0.62, 0.96)	0.64 (0.45, 0.91)	0.003	0.471

CKD, chronic kidney disease; MVD, microvascular disease. Model 1 was adjusted for age (continuous, years), sex, ethnicity (White, Asian or Asian British, Black or Black British, and mixed), and Townsend deprivation index (in quintile). Model 2 was further adjusted for BMI (continuous, kg/m^2^), physical activity (continuous, MET-h/week), alcohol consumption (never, former, current: <1, 1–2, and ≥3 drinks/week), and diet score (continuous, points). Model 3 included all covariates from Model 2, plus an additional adjustment for hypertension (yes, no), hyperlipidemia (yes, no), HbA1c levels (continuous, mmol/mol), and diabetes duration (continuous, years).

## Data Availability

UK Biobank data can be requested by bona fide researchers for approved projects, including replication (https://www.ukbiobank.ac.uk/, accessed on 3 March 2023).

## Supplementary Materials

The following supporting information can be downloaded at https://www.mdpi.com/article/10.3390/nu15183910/s1. Table S1: Definitions of CVD and MVD and the associated data source. Table S2: Hazard ratios of cardio- and microvascular diseases for coffee consumers (vs. non-consumers) with different preference of coffee types. Figure S1: Flowchart of the selection of the study population from the UK Biobank study. Figure S2: Subgroup analyses for the associations of coffee consumption with risk of total cardiovascular disease (2–4 vs. no cups/day) and CKD (per 2 cups per day). Figure S3: Hazard ratios of total cardio- and microvascular disease associated with different levels of coffee intake (vs. no coffee consumption) for participants usually consuming decaffeinated, instant, or ground coffee.
