# Supplementary material for: Coffee Consumption and Incidence of Cardiovascular and Microvascular Diseases in Never-Smoking Adults with Type 2 Diabetes Mellitus
Source: Nutrients. 2023 Sep 8;15(18):3910. doi: 10.3390/nu15183910 (PMC10535817; doi:10.3390/nu15183910)
Supplement: Supplementary file 1 [file nutrients-15-03910-s001.zip › nutrients-2574303-supplementary.pdf]

Supplementary Table S1. Definitions of CVD and MVD and the associated data source.

Cardiovascular and microvascular outcomes	ICD-10
CHD	I20–I25
MI	I21-I22
stroke	I60, I61, I62.9, I63, I64, I67.8, I69.0, and I69.3
HF	I50
Retinopathy	E10.3, E11.3, E12.3, E13.3, E14.3, H28.0, H33, H35.3, H36.0, and H40-H42
Neuropathy	E10.4, E11.4, E13.4, E14.4, G59.0, G62.9, G63.2, and G99.0
CKD	E10.2, E11.2, E13.2, E14.2, N18-N19, N28.0, and I70.1

CHD, coronary heart disease; CKD, chronic kidney disease; CVD, cardiovascular disease; HF, heart failure; MI, myocardial infarction. MVD, microvascular disease; ICD, International Classification of Disease.

Supplementary Table S2. Hazard ratios of cardio-and microvascular diseases for coffee consumers (vs. non-consumers) with different preference of coffee types.

	**None**	**Decaffeinated coffee**			**Instant coffee**			**Ground coffee**		
	1.00 (Ref.)	**0.5-1**	**2-4**	**≥5**	**0.5-1**	**2-4**	**≥5**	**0.5-1**	**2-4**	**≥5**
**Total CVD**										
Events/N	548/2,859	114/578	139/760	35/188	295/1,520	331/1,930	117/571	80/507	104/626	24/99
Model 3	1.00 (Ref.)	1.00 (0.81, 1.24)	0.93 (0.76, 1.12)	0.79 (0.54, 1.14)	0.87 (0.75, 1.01)	0.80 (0.69, 0.92)	0.89 (0.72,1.10)	0.82 (0.64, 1.05)	0.77 (0.62, 0.96)	0.88 (0.56, 1.38)
**CHD**										
Events/N	412/2,859	92/578	102/760	28/188	229/1,520	255/1,930	85/571	61/507	78/626	15/99
Model 3	1.00 (Ref.)	1.08 (0.85, 1.37)	0.89 (0.71, 1.11)	0.80 (0.52, 1.22)	0.90 (0.76, 1.07)	0.82 (0.70, 0.97)	0.87 (0.68, 1.12)	0.80 (0.60, 1.06)	0.77 (0.60, 0.98)	0.70 (0.39, 1.25)
**MI**										
Events/N	174/2859	29/578	27/760	13/188	89/1,520	99/1,930	39/571	24/507	29/626	7/99
Model 3	1.00 (Ref.)	0.92 (0.62, 1.36)	0.63 (0.42, 0.95)	0.88 (0.47, 1.68)	0.87 (0.66, 1.14)	0.82 (0.63, 1.06)	1.04 (0.72, 1.50)	0.77 (0.49, 1.22)	0.72 (0.47, 1.09)	1.06 (0.49, 2.27)
**Stroke**										
Events/N	100/2,859	14/578	25/760	6/188	58/1,520	53/1,930	23/571	13/507	19/626	7/99
Model 3	1.00 (Ref.)	0.70 (0.40, 1.23)	0.89 (0.56, 1.41)	0.93 (0.40, 2.12)	0.88 (0.62, 1.24)	0.70 (0.49, 0.99)	0.87 (0.52, 1.45)	0.75 (0.41, 1.37)	0.82 (0.50, 1.37)	1.69 (0.74, 3.90)
**HF**										
Events/N	177/2,859	30/578	32/760	11/188	78/1,520	101/1,930	29/571	26/507	26/626	11/99
Model 3	1.00 (Ref.)	0.81 (0.55, 1.21)	0.66 (0.45, 0.97)	0.87 (0.47, 1.60)	0.74 (0.56, 0.98)	0.72 (0.56, 0.93)	0.65 (0.43, 0.98)	0.92 (0.61, 1.39)	0.58 (0.38, 0.89)	1.21 (0.61, 2.37)
**Total MVD**										
Events/N	426/2,191	85/443	89/603	28/155	213/1,191	275/1,511	77/448	63/403	84/502	14/81
Model 3	1.00 (Ref.)	0.99 (0.78, 1.23)	0.71 (0.56, 0.90)	0.82 (0.55, 1.23)	0.87 (0.73, 1.03)	0.85 (0.72, 0.99)	0.79 (0.61, 1.02)	0.80 (0.60, 1.06)	0.84 (0.66, 1.07)	0.87 (0.50, 1.51)
**Retinopathy**										
Events/N	230/2,191	50/443	47/603	19/155	122/1,191	159/1,511	50/448	34/403	53/502	8/81
Model 3	1.00 (Ref.)	1.10 (0.80, 1.51)	0.74 (0.53, 1.02)	1.15 (0.71, 1.87)	0.96 (0.77, 1.21)	0.93 (0.75, 1.15)	1.02 (0.75, 1.40)	0.75 (0.50, 1.11)	1.02 (0.75, 1.40)	0.92 (0.43, 1.95)
**Neuropathy**										
Events/N	64/2,191	9/443	14/603	5/155	23/1,191	42/1,511	6/448	12/403	10/502	3/81
Model 3	1.00 (Ref.)	0.83 (0.41, 1.69)	0.81 (0.43, 1.52)	1.11 (0.44, 2.78)	0.67 (0.41, 1.11)	0.91 (0.59, 1.38)	0.44 (0.19, 1.02)	1.06 (0.53, 2.09)	0.74 (0.38, 1.47)	1.36 (0.42, 4.39)
**CKD**										
Events/N	192/2,191	41/443	46/603	7/155	103/1,191	124/1,511	34/448	30/403	29/502	5/81
Model 3	1.00 (Ref.)	1.02 (0.72, 1.45)	0.77 (0.55, 1.08)	0.34 (0.14, 0.82)	0.88 (0.68, 1.13)	0.82 (0.65, 1.05)	0.74 (0.50, 1.08)	0.92 (0.62, 1.37)	0.61 (0.41, 0.92)	0.72 (0.29, 1.75)

CHD, coronary heart disease; CKD, chronic kidney disease; CVD, cardiovascular disease; HF, heart failure; MI, myocardial infarction. MVD, microvascular disease.

Model 3 was further adjusted for age (continuous, years), sex, ethnicity (White, Asian or Asian British, Black or Black British, and mixed), Townsend deprivation index (in quintile), BMI (continuous, kg/m^2), physical activity (continuous, MET-h/week), alcohol consumption (never, former, current: <1, 1-2, and ≥3 drinks/week), diet score (continuous, points), hypertension (yes, no), hyperlipidemia (yes, no), HbA1c levels (continuous, mmol/mol), and diabetes duration (continuous, years).

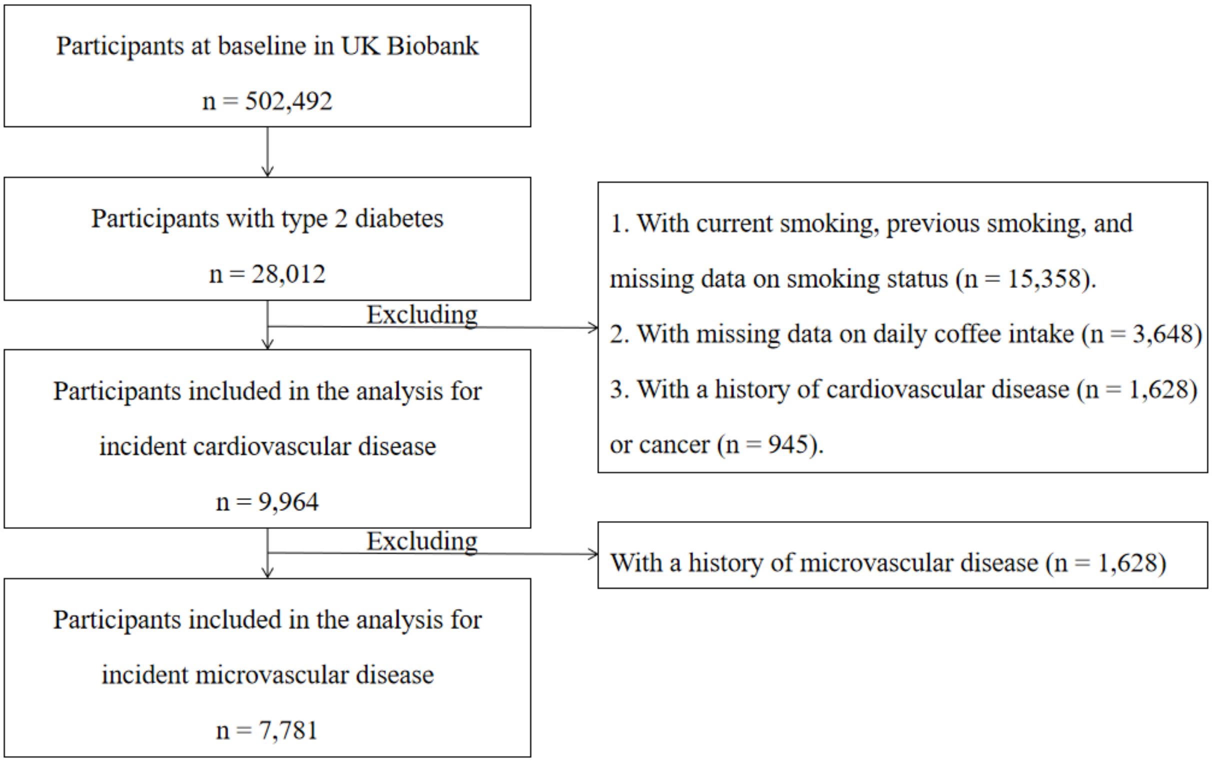

Supplementary Figure S1. Flowchart of the selection of the study population from the UK Biobank study.

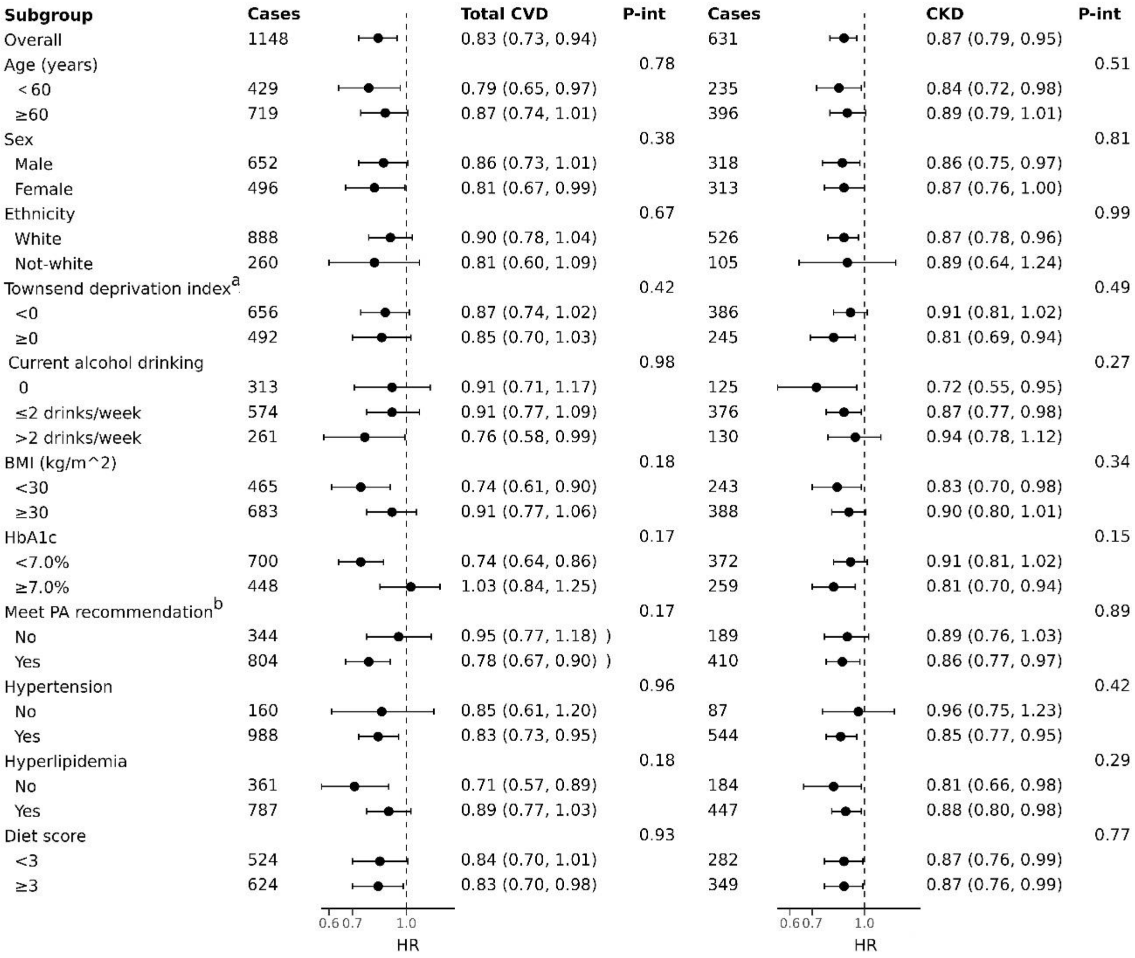

Supplementary Figure S2. Subgroup analyses for the associations of coffee consumption with risk of total cardiovascular disease (2-4 vs. no cups/day) and CKD (per 2 cups per day).

BMI, body mass index; PA, physical activity; P-int, P value for interaction.

[a] A higher Townsend deprivation index indicates a greater degree of deprivation (or lower socioeconomic status).

[b] At or >150 min/week of moderate activity or 75 min/week of vigorous activity or an equivalent combination.

Results were adjusted for age (continuous, years), sex, ethnicity (White, Asian or Asian British, Black or Black British, and mixed), Townsend deprivation index (in quintile), BMI (continuous, kg/m^2), physical activity (continuous, MET-h/week), alcohol consumption (never, former, current: <1, 1-2, and ≥3 drinks/week), diet score (continuous, points), hypertension (yes, no), hyperlipidemia (yes, no), HbA1c levels (continuous, mmol/mol), and diabetes duration (continuous, years).

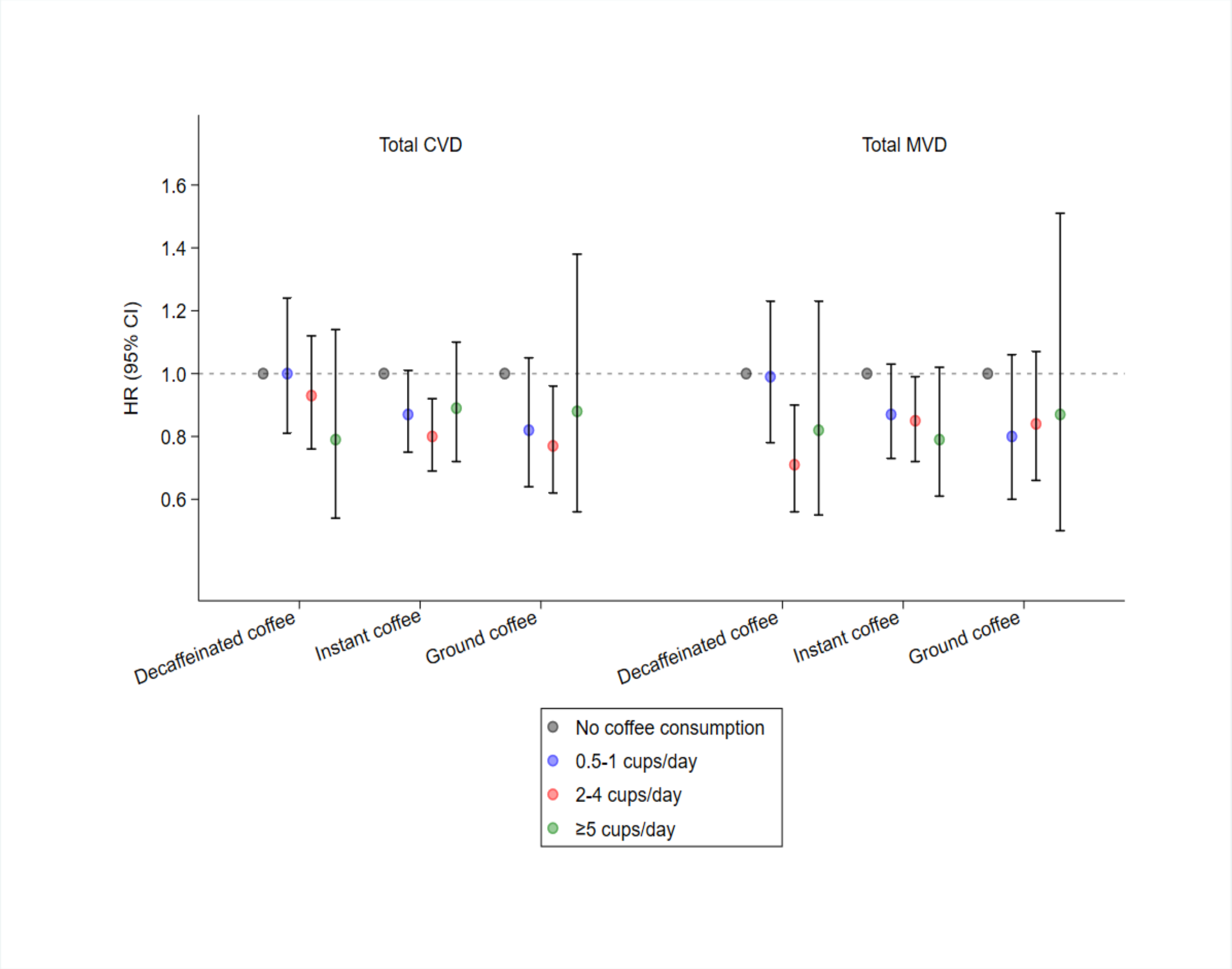

Supplementary Figure S3. Hazard ratios of total cardio- and microvascular disease associated with different levels of coffee intake (vs. no coffee consumption) for participants usually consuming decaffeinated, instant, or ground coffee.

CVD, cardiovascular disease; MVD, microvascular disease.

Results were adjusted for age (continuous, years), sex, ethnicity (White, Asian or Asian British, Black or Black British, and mixed), Townsend deprivation index (in quintile), BMI (continuous, kg/m^2), physical activity (continuous, MET-h/week), alcohol consumption (never, former, current: <1, 1-2, and ≥3 drinks/week), diet score (continuous, points), hypertension (yes, no), hyperlipidemia (yes, no), HbA1c levels (continuous, mmol/mol), and diabetes duration (continuous, years).
